# Risk Factors and Rates of Hepatitis B Virus Infection among Municipal Waste Management Workers and Scavengers in Ilorin, Kwara State, Nigeria

**DOI:** 10.5696/2156-9614-6.12.1

**Published:** 2016-12-22

**Authors:** Henry O. Sawyerr, Rauf O. Yusuf, Adedotun T. Adeolu

**Affiliations:** 1 School of Allied Health, and Environmental Sciences, College of Pure and Applied Sciences, Kwara State University, Malete, Nigeria; 2 Centre for Ecological and Environmental Research Management Studies, Kwara State University, Malete, Nigeria

**Keywords:** hepatitis B virus, municipal waste management workers, scavengers, HBV, Ilorin metropolis, Nigeria

## Abstract

**Background.:**

Poor municipal waste management, including waste treatment and disposal methods, threatens the environment and public health in most developing countries. Lack of proper municipal waste segregation and transportation techniques has increased the potential for the transmission of pathogens such as hepatitis B virus (HBV).

**Objectives.:**

This study addressed issues relating to the potential risk of infectious diseases and prevalence of HBV among municipal waste workers and scavengers in Ilorin metropolis, Nigeria.

**Methods.:**

A cross-sectional study was conducted among municipal waste management workers and waste scavengers in Ilorin metropolis, Kwara State, Nigeria. A total of 120 respondents were administered questionnaires during the first stage of the study and participated in the second (testing) stage of the study. The prevalence of an HBV infection biological marker, the Australia antigen (HBsAg), and its association with exposure to waste, socio-demographic factors, and history of occupational injuries with sharp objects/needle sticks was examined.

**Results.:**

The prevalence of HBV infection among municipal waste management workers and waste scavengers was 2.6% and 16.67% respectively, indicating that scavengers were at higher risk of HBV infection.

**Conclusions.:**

Lack of proper occupational health safety management among municipal waste management workers was a possible risk factor for HBV infection through injury with sharp instruments. The possible pathway of virus transmission was waste segregation, which is usually carried out with bare hands, and lack of hygiene and occupational safety during waste management activities. Therefore, vaccination against HBV, personal hygiene practices and regular training on occupational safety will help to control risk of HBV infection among municipal waste workers and scavengers.

## Introduction

Efficient waste management is a major challenge in developing countries, and Nigeria is no exception. The poor state of waste management in Nigeria is a result of inadequate facilities, poor funding, and poor implementation of policies, as well as changes in lifestyle, economic development, urbanization, and improved living standards.^1^ Municipal wastes are mixed and unsegregated at points of generation, undermining effective management when it comes to treatment and disposal practices. Indiscriminate disposal and sorting of mixed waste has the potential for increased environmental exposure to air pollution and toxic emissions from combusted or burnt municipal waste, and attraction and proliferation of vermin.^2^ The hepatitis B virus (HBV) is especially involved in occupational percutaneous exposures of health care workers. The risk of infection is increased by accidents at work and for HBV it is estimated to be four times greater than that of the general adult population, among those who do not work in healthcare institutions.^3^

For the purposes of this study:

- ***Municipal waste management worker***s are people employed by a public or private enterprise to collect and remove waste and recyclables from residential, commercial, industrial or other collection sites for further processing and disposal to sanitary landfills. They are trained and provided with necessary personal protective equipment (PPE) by the government or the private firm that hired them. After the training, the successful trainees are then certified with a permit.
- ***Waste scavengers, or scavengers*** are people who salvage reusable or recyclable materials thrown away by others for personal consumption or economic gain. Scavengers are unlicensed and work at dumpsites and landfills, on the streets and also visit households to collect used items. They sell what they collect to recycling facilities.

**Abbreviations**
*HbsAg*: Australia antigen
*HBV*: Hepatitis B virus
*PPE*: Personal Protective Equipment

Solid waste collection involves use of non-designated waste collection vehicles, congestion of traffic through disposal sites, and poor sorting at the source. All waste workers (both municipal waste management workers and scavengers) are exposed to a number of pathogens (bacteria, fungi, viruses, parasites and cysts), toxic substances that come from the generated waste and its decomposition. As a result of exposure to these multiple health risk factors, all waste workers suffer high rates of occupational health hazards.^4^

The health impacts of solid waste generated from households, municipalities, industry, and most importantly, healthcare and hospital facilities on all waste workers cannot be overemphasized. Illegal, uncontrolled and poor disposal of waste threatens the public health of all waste workers, and leads to frequent outbreaks of typhoid, diarrhea, cholera, and hepatitis A and B. Additionally, there is particular concern about the possibility of infection with human immunodeficiency virus (HIV) and hepatitis viruses B and C, for which there is strong evidence of transmission via healthcare waste. These viruses are generally transmitted through injuries from sharps and other materials contaminated by human blood. The health status of all waste workers is therefore a public health concern, as they could be potential pathways for the transmission of various communicable diseases to the general public.^5^ In addition, scavengers are also exposed to increased occupational health and safety risks as a result of unsafe handling of waste materials and lack of PPE.^6^

In Nigeria, international policy stipulating that the generator of waste is responsible for its proper management has not yet been implemented. The notion that waste is the responsibility of government authorities has meant that waste generators do not appreciate the negative impact of improper waste disposal on public health and the environment.^1^ Although waste is generated from many daily activities, biomedical waste is of great concern due to its hazardous nature and the possibility of disease transmission.^7^ However, the practice of non-segregation may increase the costs of final disposal, because infectious and non-infectious wastes may be mixed up and the wastes that could be disposed of in a landfill may require incineration, thus increasing the risks and costs of waste management. As a result of a lack of waste segregation practices in most hospitals, many hazardous materials are flushed down waste water drains that flow directly to open sewers or rivers, are mixed into general solid waste for disposal in municipal bins, or are mixed into wastes which are incinerated as potentially infectious waste.^8^

Hepatitis B is endemic throughout the world, especially in tropical and developing countries. About 300 million people worldwide are estimated to be infected with HBV. Nigeria is one of the countries with the highest incidence, with a prevalence of 10–15%.^9^ In general, prevalence is lower in areas with a high standard of living. Infection with HBV induces a spectrum of clinical manifestations ranging from mild in apparent disease to severe chronic liver diseases, which in some cases can lead to cirrhosis and carcinoma of the liver.^10^ The complex antigen found on the surface of HBV is called HBsAg (Australia antigen). The presence of HBsAg in serum or plasma is an indication of an active HBV infection, either acute or chronic. In a typical HBV infection, HBsAg will be detected 2 to 4 weeks before the alanine transaminase level becomes abnormal, and 3 to 5 weeks before symptoms or jaundice develop.

Cointreau reported that there has been little study on the health and incidence of injury in waste workers, in most developing countries, including Nigeria.^11^ Municipal waste management workers and scavengers working in dumpsites in Nigeria suffer from health consequences such as frequent fever, malaria, body aches, cuts and bruises and general weakness.^12^ They also live in poor housing conditions, often lacking access to a safe water supply and sanitation.^13^ They are often unaware of the dangers, but are economically dependent on their occupation for their livelihood. Ilorin metropolis is no exception with regard to the problems associated with waste disposal and its resultant health effect on waste workers. Therefore, this study was designed to assess the prevalence of viral HBV infection and virus exposure routes among municipal waste management workers and scavengers in Ilorin metropolis.

## Methods

### Study Area

The city of Ilorin lies on latitude North 8° 30′ and longitude East 4° 35′ near the southern fringe of the savannah and forest zone. It had a population of 777,667 in the 2006 census. It is surrounded by a wall about 10 miles in circumference and as high as 20 feet in some places. A large part of the province is located on grass plains with undulating landscapes which are well watered and highly agricultural. By the southern Nigeria provincial borders, at an elevation of 1,500 feet, there is a water hed with a river generally running from west to east and flowing into the River Niger. The ecology of the region plays an important role in people's decisions to settle in a particular area. It has a mean annual rainfall of 1,318 mm (51.9 inches), which allow inhabitants to practice arable farming. The mild climate has also attracted northern pastoralists to the region. Ilorin city is the commercial and administrative center of Kwara State. It is made up of three local government areas.

### Study Population

The study population was comprised of municipal waste management workers of private waste managers and the Kwara Waste Management Corporation, as well as scavengers working at dumpsites in Ilorin Metropolis.

### Sampling Techniques

Municipal waste management workers were selected from registered private waste managers registered with the Kwara State Environmental Protection Agency. Out of the 125 municipal waste management worker respondents, only 78 consented to participate in the study. Additionally, waste scavengers (42) were selected from the three registered dumpsites within the metropolis. Therefore, 120 respondents were administered questionnaires during the first stage of the study and participated in the second stage (testing) of the study.

### Sample Collection

Blood samples were collected with the help of a medical laboratory scientist from respondents at three dumpsites in Ilorin Metropolis using intravenous needles. The needles and syringes used for the collection of blood samples were dried and sterile. Three (3) ml of venous blood was aseptically drawn from the antecubital vein of participants into a plain bottle and allowed to clot at room temperature; the sample was then spun for 5 minutes at 2500 rpm in a bench centrifuge to obtain serum. The serum obtained was tested for HBsAg antibodies using a Diaspot rapid diagnostic test strip. The rapid diagnostics test performed was based on the immune chromatographic principle and its accuracy was described by Khuroo MS.^14^

### Rapid Diagnostics Test

The Diaspot rapid diagnostic test is used to qualitatively detect the presence of HBsAg in serum or plasma specimens. The test utilizes a combination of monoclonal and polyclonal antibodies to selectively detect elevated levels of HBsAg in serum or plasma. The membrane is pre-coated with anti-HBsAg antibodies on the test line region. During testing, the serum or plasma specimen reacts with the particles coated with anti-HBsAg antibody. The mixture migrates upward on the membrane chromatographically by capillary action to react with anti-HBsAg antibodies on the membrane and generate a colored line. The presence of the colored line in the test region indicates a positive result, while its absence indicates a negative result. To serve as a procedural control, a colored line will always appear in the control line region indicating that the proper volume of specimen has been added and membrane wicking has occurred.

**Figure 1:**
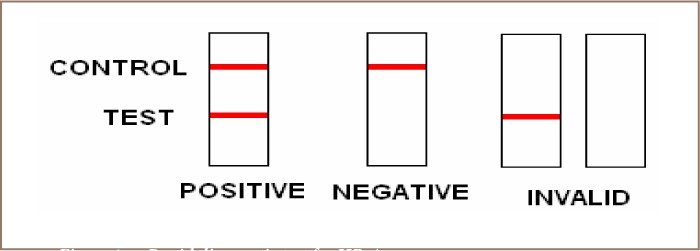
Rapid diagnostic test for HBsAg

The manufacturers' instructions were strictly followed in the performance of these tests. The test strips, serum or plasma specimens were allowed to equilibrate to room temperature (15–30° C) prior to testing. The test device was placed on a clean, level surface and 60 μl of serum or plasma was added to the sample well of the device. The sample rehydrated and was mixed with the red colloidal gold conjugate, which flowed into the membrane. After 10–15 minutes, red line(s) appeared which were read for the results of the test (*Figure 1*).

### Data Instrument

A semi-structured, interviewer-administered questionnaire was used to elicit information on the socio-demographics and occupational hazards (blood transfusion and tattoos/scarification) of all respondents. The instrument was pre-tested using 12 municipal waste management workers and scavengers from a group similar to the main study group. The Cronbach's alpha reliability test coefficient obtained was 0.64, which signified good reliability. Each question was translated into the local languages (i.e. Yoruba and Hausa) for those that could not read English, to help the respondents to give true and accurate answers.

### Data Analysis

Data were analyzed using the Statistical Package for Social Sciences, version 20. The data were analyzed using descriptive statistics such as mean and standard deviation. The chi-square (χ^2^) test was used for univariate analysis for the comparison of the prevalence of HBV markers in study subgroups.

### Ethical Approval

Ethical clearance for this study was obtained from the Kwara State Ministry of Health Ethical Review Committee. Permission was also granted by the Kwara State Environmental Protection Agency that oversees issues relating to the environment and public health in the state, and that oversees municipal waste management workers. Informed consent was obtained from each respondent before administration of the questionnaires and testing, having clearly stated that participation in the study is voluntary and individuals may decide not to further participate in the research at any time.

## Results

Table 1 shows demographic information for all respondents.

**Table 1:** Respondent Demographics

***Variables***	**Frequencies**	**Percentage (%)**
***Sex***		
**Male**	74	61.7
**Female**	46	38.3
***Age***		
**10–19 years**	7	5.8
**20–29 years**	29	24.2
**30–39 years**	53	44.2
**40–above**	31	25.8
***Marital status***		
**Single**	20	16.7
**Married**	100	83.3
***Occupation***		
**Municipal waste management workers**	78	65.0
**Scavengers**	42	35.0
***Educational Level***		
**None**	32	26.7
**Primary**	46	38.3
**Secondary**	36	30.0
**Tertiary**	6	5.0

Table 2 outlines results for risk factors and shows that a few respondents (8.3%) had undergone blood transfusion at least once in their lifetime. In addition, more than half (51.7%) of the respondents had either received a tattoo or undergone scarification.

**Table 2:** Exposure of Respondents to Risk Factors

	**Number**	**Percentage (%)**
***Blood Transfusion***		
**Received**	10	8.3
**Never received**	110	91.7
***Tattoos/Scarification***		
**Yes**	62	51.7
**No**	58	48.3
***Use of Personal Protective Equipment***		
**Always**	10	8.3
**Never**	110	91.7

The majority (91.7%) of the respondents did not use PPE. The analysis shown in Table 3 suggests that there were no significant differences between the ages of the respondents and the prevalence of HBV. There were significant differences between education level, use of PPE, occupation and the prevalence of HBV among the respondents.

**Table 3:** Prevalence of Hepatitis B Virus Infection Among Respondents

	**Frequencies**	**Positive**	**Negative**	**Chi-square**
***Age***				
**10–19 years**	7	2	5	
**20–29 years**	29	1	28	5.560+
**30–39 years**	53	3	50	
**40–Above**	31	3	28	
***Education Level***				
**None**	32	2	30	
**Primary**	46	4	42	0.694^***^
**Secondary**	36	3	33	
**Tertiary**	06	-	6	
***Use of PPE***				
**Always**	10	2	8	2.46^***^
**Never**	110	7	103	
***Occupation***				
**Municipal waste management workers**	78	2 (2.6)	76 (97.4)	11.47^***^
**Scavengers**	42	7(16.7)	35 (83.3)	

Values in parenthesis are percentages

+, p>0.05

***, p<0.05

## Discussion

The risk of HBV infection increased with increasing age and longer duration of employment among the municipal staff, which might be due to the higher chance of exposure to risk factors and different sources of infection over time. This result was similar to a study where the age of acquiring infection was found to be the major determinant of the incidence of HBV.^15^ The age distribution of the study indicated that young individuals (30–39 years) made up the majority of the waste worker population. This can be attributed to their agility, high level of poverty, lack of parental care and high level of unemployment. On the other hand, there was an inverse association between educational status and HBsAg positivity, with less educated respondents showing the highest level of positivity, indicating the positive influence of education and public enlightenment/awareness on the carrier rate of HBV infection and transmission routes. It is worthy of note that the educational level of respondents was found to affect the prevalence of HBV infection.

The present study found that despite the impact of age, educational level and occupation on the occurrence of HBV infection, occurrence of blood transfusion, use of PPE and high-risk behaviors (tattoos/scarification) might be responsible for the high rates of positive HBsAg among the respondents after controlling for confounding factors.

The higher prevalence of HBV among scavengers compared to municipal waste management workers could be attributed to the nature of waste scavenging, which often involves gathering of sharp instruments, metals and infective wastes for re-selling. Handling of waste during segregation is usually improperly carried out with bare hands and there is a lack of hygiene and occupational safety. Scavengers often live on or beside dumpsites in order to await the arrival of trucks filled with waste. Waste is sorted with bare hands, sticks or simple hooks. Even a minor injury with a sharp instrument with little loss of blood carries the risk of transfer of HBV and other diseases.^16^ Scavenging activities are often carried out by poor, uneducated and unhealthy groups which nevertheless play an important role in recycling activities in developing nations such as Nigeria.^1^

Municipal waste workers and scavengers are usually exposed to injury by sharp instruments and lack of safety measures during the performance of their duties. The procedures most often performed that carry risk of injury include waste offloading, segregation, transportation of recyclable materials to the point of sale, and disposal. Minor injury with a sharp instrument, even with little loss of blood, carries the risk of transferring diseases such as hepatitis, HIV/AIDS, brucellosis and others.^16^,^17^ HBV infection is a serious worldwide public health problem and presents a significant biological risk to health care workers, although other occupational groups such as public safety workers, military forces and sewage workers are also at increased risk. Participants in this study who tested positive were advised to seek medical attention and scavengers were advised to register with the local authority to train and obtain a permit to scavenge waste.

## Conclusions

The present study suggests that there is a higher prevalence of HBV infection for waste scavengers than the general population, indicating possible pathways of virus transmission through waste segregation activities, which are usually carried out with bare hands, and a lack of hygiene and occupational safety during waste management activities. It is important to provide all waste workers with PPE and thorough instructions on its use. In addition to vaccination against HBV, educational campaigns and regular training on occupational health and safety programs and health surveillance should be instituted for all waste workers with an emphasis on good work practices, immunization and personal hygiene practices, as this is essential to the control of HBV infection among members of this occupational group. Solid waste management policies and enforcement of sanitation laws should be institutionalized, and environmental organizations have important roles to play in efforts to make a clean environment in Nigeria a reality.
